# Targeting fibroblasts in immune mediated inflammatory diseases: a cellular basis for cure?

**DOI:** 10.1093/rheumatology/keag249

**Published:** 2026-05-11

**Authors:** Mikalena Xenophontos, Fränze Progatzky, Christopher D Buckley

**Affiliations:** Kennedy Institute of Rheumatology, University of Oxford, Oxford, UK; Kennedy Institute of Rheumatology, University of Oxford, Oxford, UK; Kennedy Institute of Rheumatology, University of Oxford, Oxford, UK

**Keywords:** fibroblast, rheumatoid arthritis, synovium, animal models, cytokines and inflammatory mediators, inflammation, metalloproteinases

## Abstract

Fibroblasts constitute a major component of our stroma, the supportive bedding in which our tissues reside. The introduction of advanced single-cell technologies has greatly enhanced our appreciation of fibroblast heterogeneity in health and immune-mediated inflammatory diseases (IMIDs). This heterogeneity correlates with their diverse functions, including providing architectural support, tissue identity, ‘stromal memory’, and regulating immune responses and fibrosis. In RA, fibroblasts contribute to both synovial inflammation and bone and cartilage damage, and in IBD to loss of the epithelial barrier, intestinal inflammation, and the development of intestinal strictures and fistulae in Crohn’s disease. Fibroblasts have also been associated with non-response to current biologic therapies in RA and IBD. Targeting pathogenic fibroblast populations using new therapeutic modalities such as Chimeric Antigen Receptor (CAR) T-cell therapies may ‘reset’ the stroma back to health and give hope for cure in these debilitating IMIDs.

## Graphical abstract

**Figure:**
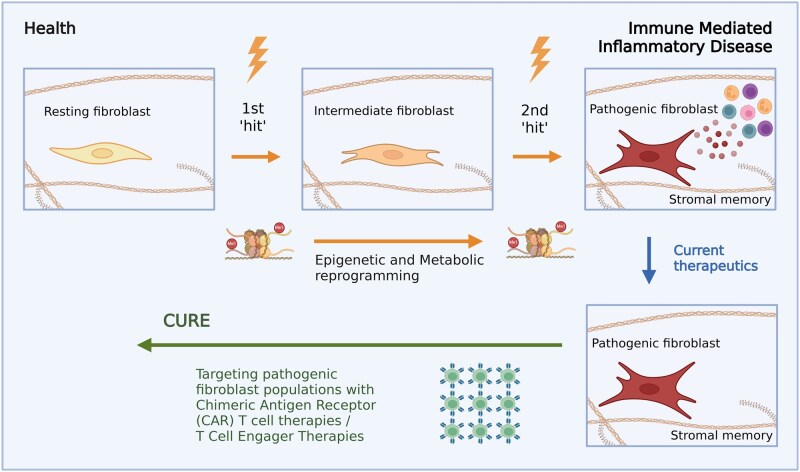
Figure created using BioRender

Rheumatology key messages

- Single-cell technologies have uncovered the transcriptional and functional heterogeneity of fibroblasts in health and immune-mediated inflammatory diseases (IMIDs).
- Pathogenic fibroblast populations regulate immune responses and/or fibrosis in IMIDs .
- Targeting pathogenic fibroblast populations in IMIDs may restore stromal integrity and offer hope for curative therapies for these debilitating disorders.

## Introduction

Have you ever wondered what determines the specific sites of inflammation in the human body? Why does arthritis affect the joints but not the colon, while colitis occurs in the colon and spares the skin, and why does psoriasis develop in the skin rather than the liver? The answer to these questions lies at the heart of why rheumatologists and their patients should be interested in fibroblasts. If you want to know more about these cells, their function in RA and IBD and why targeting them therapeutically gives a glimpse of hope to curing immune-mediated inflammatory diseases (IMIDs), then read on.

What are fibroblasts? Fibroblasts were first described in 1858 by Rudolf Virchow as ‘spindle-shaped cells of the connective tissue’ [1]. They are still defined today by their spindle shaped morphology, absence of leucocyte, endothelium and epithelium lineage markers and ability to bind to plastic [2]. They are mostly thought to originate from the embryonic mesenchyme, which is derived from the embryonic mesoderm, although they can also be derived from the process of epithelial to mesenchymal transition [3, 4] or from circulating fibrocytes thought to derive from peripheral monocytes [5]. Fibroblasts in the head and neck region also partly originate from the neural crest [6].

Fibroblasts are a major component of our stroma which as its name suggests, provides the ‘bedding’ in which tissues lie. Stroma is composed of connective tissue, nerves, vessels and extracellular matrix (ECM). Fibroblasts produce collagens, proteoglycans, elastin, fibronectin, microfibrillar proteins and laminins which form the ECM. They also remodel the ECM by producing MMPs, which are the enzymes responsible for ECM degradation, and MMP inhibitors [7].

## The transcriptional and functional heterogeneity of fibroblasts

**Figure 1:**
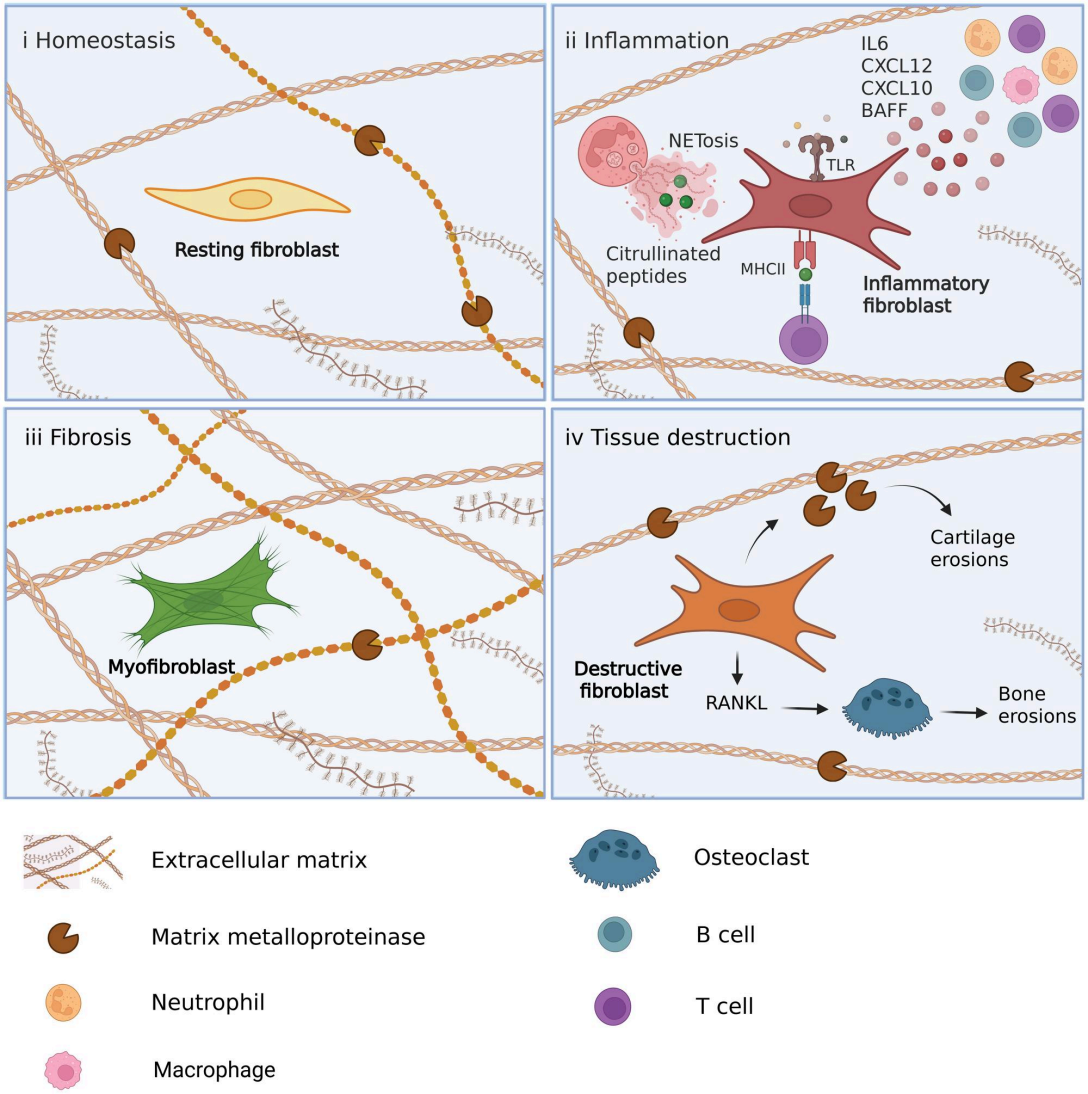
Functional heterogeneity of fibroblasts in health and disease. (i) In homeostasis, fibroblasts maintain structural tissue integrity by balancing extracellular matrix synthesis and degradation (via MMPs). (ii) In inflammation, fibroblasts adopt a pro-inflammatory phenotype and facilitate immune responses by secreting chemokines and cytokines which attract and activate immune cells, present antigen via MHC class II and respond to inflammatory stimuli through the expression of pattern recognition receptors such as Toll-like receptors (TLRs). In RA, fibroblasts can present citrullinated peptides derived from neutrophil extracellular traps (NETs) to T cells. (iii) Fibroblasts may differentiate into contractile myofibroblasts, which in the presence of chronic injury and inflammation can lead to the deposition of excessive and disorganized extracellular matrix, and ultimately lead to fibrosis. (iv) Fibroblasts can adopt a ‘destructive’ phenotype which is characterized by excessive production of MMPs and receptor activator of nuclear factor kappa B ligand (RANKL) which leads to cartilage erosions and bone erosions (via the effect on osteoclasts) in RA. Figure created using BioRender

In homeostasis, fibroblasts maintain a fine balance of ECM production and degradation to maintain the structural integrity of our tissues (Fig. 1). This balance can become dysregulated in disease with overproduction of disorganized ECM leading to fibrosis or excessive degradation of ECM by MMPs leading to tissue destruction (Fig. 1). Fibroblasts can adopt the contractile phenotype of a myofibroblast, a cell that resembles smooth muscle [8]. Myofibroblasts play a vital role in repair (including wound repair [9]) as they secrete and remodel the ECM [10]. However, in the presence of chronic injury and inflammation they can produce excessive and disorganized ECM which ultimately leads to the accumulation of stiff and collagenous scar tissue and subsequent tissue fibrosis [11] (Fig. 1). The TGF-β signalling pathway is important for the activation of myofibroblasts [12]. Fibroblasts can also adopt a ‘destructive’ phenotype via the production of MMPs [13–15] and receptor activator of nuclear factor kappa B ligand (RANKL) [16] which can lead to cartilage and bone erosions in RA (Fig. 1).

Beyond their role in structural support, fibroblasts can facilitate immune responses via the production of chemokines and cytokines which attract and activate immune cells [17, 18], adoption of immune regulatory functions such as antigen presentation to T cells [19, 20] and expression of pattern recognition receptors such as Toll-like receptors (TLRs) which enable them to respond to inflammatory stimuli [21] (Fig. 1).

The role of mesenchymal cells in positional and tissue identity has been recognized for a long time. Traditional tissue recombination studies between birds and mammals demonstrated that fibroblasts in the dermis were responsible for determining the cell fate of the epithelium into either feathers or hair [22]. This positional identity is thought to be due to the differential expression of *HOX* genes along the craniocaudal axis [2, 23]. This ability of fibroblasts to define positional identity was also demonstrated by Frank-Bertoncelj *et al.* who showed that synovial fibroblasts from joints in different anatomical locations have unique phenotypes [24] which are driven by epigenetic changes driving *HOX* gene expression [24]. This positional identity of fibroblasts may be responsible for driving inflammation to specific anatomical sites and may explain why RA prefers the proximal to the distal IP joint and why colitis is driven to the colon and not the liver.

Another, less well-recognized function of fibroblasts is their ability to ‘recall’ prior inflammatory insults, even after apparent resolution of inflammation. Through long-lasting changes in chromatin accessibility, previous inflammatory exposure can prime fibroblasts to mount an exaggerated response to subsequent stimuli [25]. This phenomenon, which resembles trained immunity for macrophages [26, 27], is termed ‘stromal memory’ in this review and may help explain the persistence and relapse characteristic of IMIDs and will be explored further below [28].

It is now well established that fibroblasts do not represent a homogeneous cell population, but are instead transcriptionally heterogeneous, a concept that has become evident with advances in single-cell technologies such as single-cell RNA sequencing. Fibroblasts display remarkable intra-organ and inter-organ heterogeneity [29, 30] which is unsurprising given their diverse functionality. The two fibroblast markers that seem to be consistently identified on resting fibroblasts across different organs are peptidase inhibitor 16 (PI16) and collagen type 15 alpha-1 (COL15A1) [30]. PI16^+^ fibroblasts are thought to be able to develop into all specialized fibroblasts in tissues while COL15A1^+^ fibroblasts secrete basement membrane proteins. Despite their heterogeneity, two shared fibroblast states, namely the CXCL10^+^CCL19^+^ and SPARC^+^COL3A1^+^ fibroblast populations, were found across four different IMIDs; RA, IBD, Sjögren’s disease and interstitial lung disease [31]. The CXCL10^+^CCL19^+^ and perivascular SPARC^+^COL3A1^+^ populations seem to interact with immune cells and endothelial cells, respectively, and resemble the HLA^hi^ fibroblasts and NOTCH3^+^ fibroblast populations in RA (described below).

This review will discuss the role of fibroblasts in two common IMIDs: RA and IBD. We choose to discuss fibroblasts in these two IMIDs as fibroblasts are well-characterized and seem to share common parallels including their implication in non-response to treatment. This review will not discuss fibroblasts in other IMIDs or cancer-associated fibroblasts (CAFs). CAFs form an integral part of the tumour microenvironment and have established roles in driving tumorigenesis which have been extensively reviewed elsewhere [32, 33].

## Synovial fibroblasts in RA

RA, a debilitating disease of joint inflammation, pain and disability, affects up to 1% of the population. Despite the introduction of biologic and targeted synthetic therapies there remains a significant proportion of patients who do not respond to these therapies. The term ‘difficult to treat (D2T) RA’ was introduced by the EULAR taskforce in 2021 [34]. The prevalence of D2T RA is estimated to range between 5.5% [35] and 27.5% [36]. This reflects a significant clinical unmet need and highlights the need for the development of more therapeutics to achieve disease remission or even cure.

**Figure 2:**
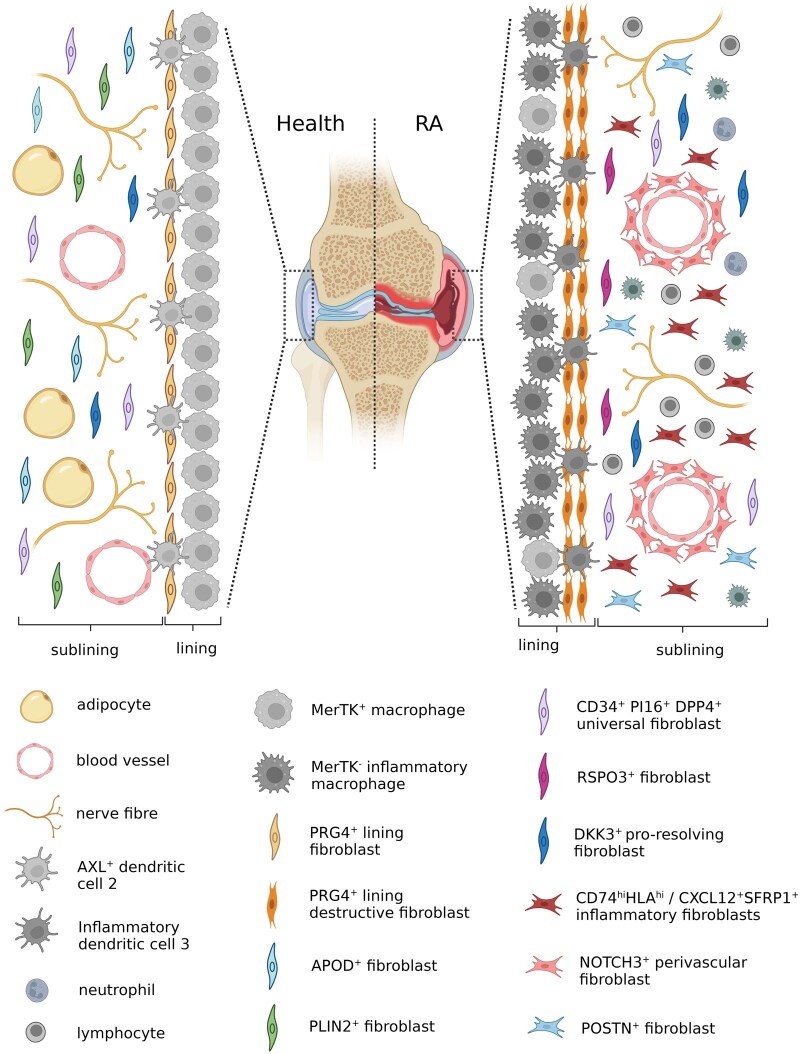
Synovial fibroblast heterogeneity in health and in RA. In health, the lining layer is composed of MerTK^+^ macrophages, AXL^+^ dendritic cell 2s and PRG4^+^ lining fibroblasts. The sublining layer is composed of homeostatic APOD^+^ and PLIN2^+^, pro-resolving DKK3^+^ and the universal CD34^+^, PI16^+^, DPP4^+^ fibroblast populations, adipocytes, blood vessels and nerve fibres. In RA there is loss of adipocytes, homeostatic APOD^+^ and PLIN2^+^ fibroblasts, MerTK^+^ lining macrophages and tolerogenic AXL^+^ type 2 dendritic cells. Lining layer PRG4^+^ fibroblasts adopt a destructive phenotype. There is expansion of sublining CD74^+^HLA^hi^, CXCL12^+^ SFRP1^+^ inflammatory fibroblasts, perivascular NOTCH3^+^ fibroblasts, POSTN^+^ fibroblasts and RSPO3^+^ fibroblasts. There is infiltration of inflammatory type 3 dendritic cells in the lining layer and of lymphocytes, neutrophils and MERTK^–^ macrophages in the sublining layer. Figure created using BioRender

Fibroblasts form a major component of the soft tissues of our joints including the synovium [37]. In health, the synovium consists of a one- to three-cell thick layer, called the synovial lining layer (LL), which is composed of proteoglycan 4 (PRG4)^+^ fibroblasts, MerTK^+^TREM2^pos^ tissue resident macrophages and tolerogenic AXL^+^ dendritic cell 2s (DC2s) [38–40] (Fig. 2). LL fibroblasts produce lubricin, also known as PRG4, which functions as a joint lubricant. The sublining layer (SL) is composed of healthy fibroblast populations including the PLIN2^+^ and APOD^+^ fibroblast clusters which are enriched in genes involved in lipid metabolism and insulin signalling, respectively [41] (Fig. 2). The SL is also composed of macrophages, ECM, adipocytes, blood vessels and nerve fibres [38, 42, 43]. In RA, there is marked expansion of the SL with loss of adipocytes, infiltration of immune cells and expansion of pathogenic fibroblast populations [41, 44] (Fig. 2). Tolerogenic AXL^+^ DC2s are also replaced with inflammatory dendritic cell 3s (DC3s) in the LL [40]. The use of US-guided synovial tissue biopsies and advanced sequencing techniques has revolutionized our understanding of the cellular components of this tissue and how it changes in disease.

### Synovial fibroblast heterogeneity in RA

Three different cellular pathotypes were originally defined histologically in the synovium in early RA, namely (i) lympho-myeloid, dominated by the presence of B cells and myeloid cells, (ii) diffuse myeloid which is dominated by macrophages and (iii) pauci-immune, dominated by stromal cells and fibroblasts with very few immune cells present [45]. A collaborative effort by the Accelerating Medicines Partnership 2 (AMP2) [46] consortium analysed 82 different synovial biopsies from patients with moderate or active disease activity at different stages of their disease and described six different cell type abundance phenotypes (CTAPs); (i) endothelial, fibroblast and myeloid cells, (ii) fibroblasts, (iii) T cells and fibroblasts, (iv) T and B cells, (v) T and myeloid cells and (vi) myeloid cells.

The fibroblast pathotypes have been implicated with non-response to therapies in RA [47]. This is likely due to the fact that current therapies have no direct effect on synovial fibroblasts. The pauci-immune pathotype was associated with non-response to treatment after 6 months of DMARD therapy in patients with early RA [48] and following treatment with the TNF inhibitor certolizumab-pegol [49]. The presence of CTAP-F pathotype combined with a fibroblast signature was also found to have the poorest response when the bulk RNA sequencing data from the R4RA [50] clinical trial comparing rituximab and tocilizumab in TNF inadequate responders was analysed [46, 47].

The AMP2 consortium identified two lining, one intermediate and six sublining fibroblast populations in the synovium of patients with RA [46] (Fig. 2). The LL populations were PRG4^high^ and the SL populations were Thy1^high^ and PRG4^low^. Thy1 is a cell surface glycoprotein that is important for mechanosensation and preventing excessive fibroblast contractility. Loss of Thy1 was found to promote myofibroblast differentiation and contribute to fibrosis in the heart and lung [51–54]. The lining fibroblast populations included the PRG4^+^CLIC5^+^ (F-0) and PRG4^+^ (F-1). The RSPO3^+^ (F-8) population is an intermediate state and has features of both the lining and sublining. The SL populations included CD34^+^ (F2), POSTN^+^ (F3), DKK3^+^ (F4), CD74^hi^HLA^hi^ (F5), CXCL12^+^SFRP1^+^ (F6) and NOTCH3^+^ (F7). The F2 cluster expressed PI16 and DPP4, pointing to a progenitor like population. The F5 and F6 clusters are the inflammatory fibroblast populations as they express CXCL12 and IL-6.

The distinct functions of lining and sublining fibroblasts was confirmed when THY1^−^Fibroblast-activation protein (FAP)α^+^ and THY1^+^FAPα^+^ fibroblasts were adoptively transferred into the joints of mice with arthritis. LL THY1^−^FAPα^+^ fibroblasts contributed to bone and cartilage damage, whereas the THY1^+^FAPα^+^ cells in the SL contributed to a more severe and persistent inflammatory arthritis [13]. Moreover, genetic deletion of FAPα^+^ fibroblasts reduced inflammation and bone erosions in a mouse model of arthritis [13]. Endothelium-derived NOTCH3 signalling was found to drive the expansion of the perivascular THY1^+^ fibroblast population and provide positional identity to fibroblasts in the synovium [55].

Aside from promoting inflammation, a subpopulation of fibroblasts marked by the expression of CD200^+^DKK3^+^ has recently been associated with resolving inflammation [56]. In an exciting experimental medicine study PET scanning was used to detect FAP tracer uptake as a marker of fibroblast activation in both humans and mice. Mice overexpressing human TNF or mouse IL-23 showed a reduction in tracer uptake in joints following TNF and IL-17 inhibition. This reduction in FAP uptake was attributed to a switch from pro-inflammatory MMP3^+^/IL-6^+^ fibroblasts to pro-resolving CD200^+^DKK3^+^ fibroblasts [56]. Type VI collagen expression by fibroblasts has also recently been found to contribute to the resolution of inflammation by preventing ingress of immune cells from the perivascular zone [57].

The use of spatial transcriptomics which allows the study of both the molecular profile of cells but also the tissue architecture has enabled the characterization of distinct fibrogenic niches in RA synovium including the COMP^hi^ synovial fibroblast [58]. The Notch-TGF-β pathway seems to be driving this fibrogenic population of fibroblasts which has been associated with treatment non-response in RA [58].

### Synovial fibroblasts as immune regulators and tissue destructors

The role of fibroblasts in promoting inflammation and tissue destruction in RA is well characterized. These functions are thought to be mainly driven by different populations of synovial fibroblasts as described above [13]. Fibroblasts drive inflammation in many different ways, including the production of cytokines and interaction with cells of our innate and adaptive immune system. Synovial fibroblasts can be thought of as ‘cytokine factories’ producing IL-6, IL-8, G-CSF, IL-33, IL-11, IL-1α and IL-1β [17]. In fact, most of the IL-6 production in the joints is thought to derive from fibroblasts [17]. IL-6 production was found to be under the control of an autocrine loop whereby autocrine leukaemia inhibitory factor (LIF) drives the continued production of IL-6 via LIF receptor (LIFR) and STAT4 signalling [17]. Synovial fibroblasts can also recruit T cells via the production of chemokines such as CXCL10 [59]. They also act as cells of our innate immune system as they express pattern recognition receptors such as TLR3 and -4, and can produce IL-6 and MMPs in response to their activation [21]. TLR3 signalling in synovial fibroblasts has also been shown to produce BAFF and APRIL which facilitate class switching in B cells [60]. The ability of RA synovial fibroblasts to act as antigen-presenting cells and present antigen to CD4 T cells via their MHC class II receptors has also been demonstrated [19, 20]. Synovial fibroblasts can take up citrullinated peptides found in neutrophil extracellular traps (NETs) secreted by activated neutrophils and in turn present these to T cells on their MHC II receptors [61] (Fig. 1). In this way they can link the activation of the innate and adaptive immune systems in RA and contribute to the production of ACPAs, which are well known for their association with severe erosive RA [62].

Synovial fibroblasts drive bone and cartilage erosions via the production of RANKL [16] and MMPs, respectively. THY1^–^FAPα^+^ fibroblasts expressed high levels of RANKL and MMP3, MMP9 and MMP13, and caused bone and cartilage erosions when adoptively transferred in a mouse model of arthritis [13]. MMP13 and Membrane type 1 (MT1) MMP have been extensively studied *in vitro* and in mouse models of arthritis. MMP13 [15] and MT1 MMP [14] are upregulated by synovial fibroblasts at the junction between inflamed hyperproliferated synovial tissue, also known as pannus tissue, and cartilage. Inhibition of both MMP13 [63] and MT1 MMP [64] lead to reduced cartilage erosions in mouse models of arthritis. The ETS1 transcription factor is thought to be responsible in driving this pathogenic tissue destructive population of fibroblasts [65].

Cadherin-11 (CDH11) which provides adhesion between fibroblasts has been extensively studied in RA [66] and is thought to drive both inflammation and cartilage degradation. CDH11 engagement mediates inflammation by inducing the production of pro-inflammatory cytokines including IL-6 [67]. Mice with a genetic deletion in CDH11 had reduced joint inflammation and reduced cartilage erosions in a mouse model of arthritis [68]. In addition, fibroblasts in culture that lacked CDH11 showed less invasion compared with wild-type fibroblasts [68].

### Synovial fibroblasts in the generation of pain

Synovial fibroblasts have more recently been implicated in the generation of pain in patients with RA. A high inflammatory score in the synovium of patients with RA correlates with pain, however, a low inflammatory score does not [69]. Pain correlating with inflammation is unsurprising given that we know that inflammatory mediators in the joint such as IL-6 have their corresponding receptors on nociceptors in the synovium which can generate pain [70]. However, the mechanisms driving pain in the presence of low inflammation are less clear. A new study [71] used machine learning and identified an 815-gene expression module in the synovium of patients with RA that correlated with pain [71]. Most of the genes in this module were found to be expressed by LL fibroblasts, suggesting that the LL acts as the anatomical site for pain in arthritis in the presence of low inflammation.

### Epigenetic remodelling of synovial fibroblasts

Epigenetic remodelling refers to the regulation of gene expression without altering the DNA sequence itself. Common mechanisms that drive epigenetic remodelling include DNA methylation, histone modifications and non-coding RNA [72]. Environmental factors are thought to contribute to disease by driving epigenetic remodelling. Synovial fibroblasts are known to accumulate epigenetic changes. TNF stimulation of synovial fibroblasts *in vitro* led to a more prolonged expression of TNF-inducible arthritogenic genes compared to macrophages. Analysis of their epigenome identified persistent H3K27 acetylation and increased chromatin accessibility in regulatory elements associated with these arthritogenic genes [73]. Inhibiting bromodomain and extra-terminal motif (BET) proteins (which normally promote gene expression by linking histone acetylation to transcriptional machinery) *in vitro* reversed the sustained expression of these arthritogenic genes in synovial fibroblasts [73]. A recent study that analysed chromatin accessibility and gene expression of RA synovial fibroblasts identified four unique chromatin profiles (named open chromatin classes) which corresponded to multiple transcriptional cell states [74]. The S_A_−0: CXCL12+ HLA-DR^hi^ sublining fibroblast chromatin class was marked by accessibility to the *CXCL12*, *HLA-DRA* and *CD74* genes and corresponded to the following four fibroblast clusters described by the AMP 2 consortium: F-6, F-5, F-3 and F-8 [74].

Epigenetic remodelling is thought to underlie the concept of trained immunity in macrophages (referred to as ‘stromal memory’ in fibroblasts in this review). Epigenetic remodelling induces long-lasting changes in chromatin state that are more likely to persist during a second inflammatory hit [75]. The second inflammatory hit can therefore trigger a more pronounced inflammatory response. It is tempting to speculate that ‘stromal memory’ contributes to the persistent inflammation seen in IMIDs and may explain the phenomenon seen in clinical practice whereby RA is often preceded by ‘palindromic rheumatism’, a scenario where bouts of joint inflammation alternate with periods of no inflammation. This feature is also seen in some animal models of arthritis such as the K/BxN serum transfer model where repeated injection of arthritogenic serum in mice leads to a prolonged and persistent inflammatory response [13, 25]. ‘Stromal memory’ may also explain why current therapies provide no cure to IMIDs and why diseases relapse on discontinuation of therapy. Reversing these epigenetic changes that drive the formation of pathogenic fibroblast populations and restoring the stroma back to health seems imperative to providing a cure (see Graphical Abstract). Whether ‘stromal memory’ can also explain the clinical observation whereby flares of RA tend to affect the same joints [76] remains to be determined.

Epigenetic and metabolic reprogramming of THY1+ CD34+ SL fibroblasts in response to inflammation were shown to contribute to ‘stromal memory’ in this seminal paper by Friščić *et al.* [25]. Primed synovial fibroblasts were found to have increased chromatin accessibility of inflammatory, bone remodelling and metabolism genes including complement factor 3 (C3). Primed synovial fibroblasts were also found to have a different metabolic profile with a shift towards aerobic glycolysis. C3 via C3α receptor (C3αR) signalling via downstream mammalian target of rapamycin (mTOR) and hypoxia-inducible factor (HIF) 1a signalling was found to be driving the priming of synovial fibroblasts [25].

## Fibroblasts in IBD

IBD, including ulcerative colitis (UC) and Crohn’s disease (CD) are chronic relapsing–remitting IMIDs that affect millions of people worldwide [77]. UC affects the colon whereas CD can affect any part of the gastrointestinal tract from the mouth to the anus; predominantly the terminal ileum or colon. Inflammation in CD is transmural as it affects all the layers of the bowel wall whereas UC mainly affects the epithelium.

**Figure 3:**
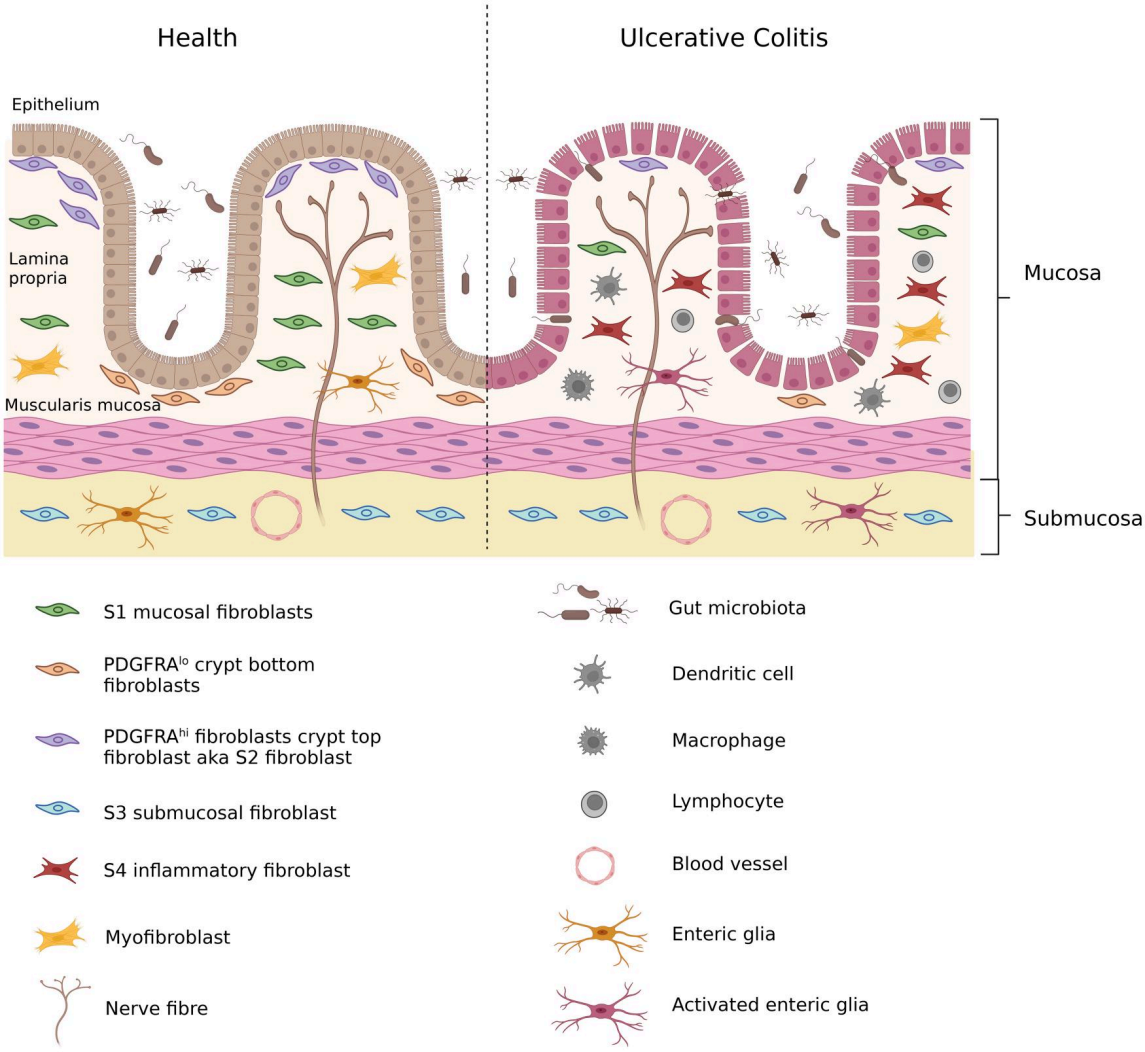
Intestinal fibroblast heterogeneity in health and in ulcerative colitis. In health, PDGFRA^lo^ fibroblasts secrete canonical Wnt signals which drive the maintenance of the stem cell niche and PDGFRA^hi^ S2 fibroblasts secrete non-canonical Wnt ligands and BMP ligands which drive epithelial cell differentiation. S1 and S3 fibroblast populations are found in the mucosa and submucosa respectively. In ulcerative colitis, there is loss of the S2 fibroblast population which contributes to disruption of the epithelial barrier and expansion of the S4 inflammatory fibroblast population which contributes to inflammation. Disruption of the epithelial barrier allows the entry of microbiota into the mucosa. This is accompanied by infiltration of lymphocytes, macrophages, dendritic cells and activation of enteric glia. Figure created using BioRender

The intestinal stem cell niche lies at the bottom of the intestinal crypts and is responsible for the continued supply of epithelial cells which undergo differentiation as they ascend the crypt villus axis [78]. Stromal cells support this niche by producing opposing gradients of Wnt and BMP signalling along the crypt-villus axis; with a high Wnt gradient at the base of the crypt to support stem cell proliferation and high BMP gradient at the top of the crypt to promote epithelial cell differentiation. Two populations of fibroblasts, namely the crypt top and crypt bottom fibroblasts, maintain epithelial cell differentiation and stem cell proliferation, respectively [79, 80]. The crypt top fibroblasts, also known as telocytes, are PDGFRA^+^ and secrete noncanonical Wnt ligands (WNT5A) and BMP ligands (BMP2/3/4/5/7). The crypt bottom fibroblasts are PDGFRA^lo^, lie close to the stem cell niche, and secrete canonical Wnt ligands (WNT2 and WNT2b) and BMP inhibitors [79, 80] (Fig. 3).

### Fibroblast heterogeneity in IBD

Heterogeneity in intestinal fibroblasts in health and IBD is recognized, however there is no agreed classification or nomenclature for these fibroblast subpopulations. Single-cell sequencing technologies identified four fibroblast subtypes in the healthy and inflamed colon of patients with UC named S1–S4 and a myofibroblast cluster [81] (Fig. 3). The S1 cluster was enriched in TNF responsive genes and was found in the lamina propria. The S2 cluster was found close to colonic epithelium and was enriched in TGF-β superfamily ligands (BMP2 and BMP5), non-canonical Wnt ligands (WNT5A and WNT5B), and the Wnt antagonist FRZB. The S2 cluster was found close to the colonic epithelium and was much smaller in patients with UC indicating disruption of the epithelial barrier. The S4 cluster was much larger in patients with UC and included genes responsible for recruiting lymphocytes such as CCL19, genes associated with fibroblastic reticular cells, the MHC class II invariant chain (CD74) and IL-33 [81]. Another study found the expansion of a subpopulation of fibroblasts referred to as inflammation-associated fibroblasts (IAFs) in patients with UC enriched for IL-11, IL-24 and IL13RA2 genes [18].

Similar to human IBD, fibroblast heterogeneity exists in health and disease in mice [81]. Using the dextran-sodium-sulfate (DSS)-induced mouse colitis model, a recent study [82] demonstrated that intestinal inflammation is accompanied by IAFs. Interestingly, IAFs persisted after resolution of inflammation and restoration of mucosal architecture, suggesting their contribution to ‘stromal memory’, though further work is needed to confirm this.

Fibroblast heterogeneity exists between different anatomical sites in CD. In particular, IL-11-producing IAFs [18] are expanded in the inflamed colon, but are not detected in the terminal ileum of patients with CD [83]. Other stromal cells such as the SMOC2^+^ PTGIS^+^ and ADAMDEC^+^ fibroblast populations were enriched in the terminal ileum and reduced in the colon of CD patients [83].

Fibroblasts also drive the development of intestinal structures and fistulae in CD. An expansion of IL-11^+^ inflammatory fibroblasts near the ulcerated epithelium and collagen^hi^ fibroblasts near the muscle and serosa was found in resection samples and biopsies from patients with CD. The collagen^hi^ fibroblasts expressed genes linked to fibrosis [84]. Mesenteric adipose tissue frequently wraps around diseased bowel in CD, a process known as creeping fat (CF). CF is often present around intestinal strictures in CD [85]. CTHRC1^+^ fibroblasts derived from CF were enriched in areas of fibrosis at the CF–bowel wall interface of intestinal strictures in patients with CD. These fibroblasts express Yes-associated protein (YAP)/transcriptional co-activator with PDZ-binding motif (TAZ) signatures which allow them to sense mechanical signals and contribute to fibrosis [86]. An additional fibroblast population has recently been identified in fistulating CD samples, named FAS cells. FAS cells have a different expression profile from FAS-like cells present at the base of ulcers. FAS cells in fistulae upregulate ECM-remodelling enzymes and developmental transcription factors which enable them to invade the tissue and form fistulae. FAS cells may also contribute to epithelialization of the fistula tract by expressing WNT5A and WNT5B, similar to telocytes [87].

### Fibroblasts are associated with non-response to therapies in IBD

Fibroblasts have also been implicated in non-response to therapies in patients with IBD. Similarly to RA, this is likely driven by the fact that current therapeutics have no direct effect on pathogenic fibroblast populations which drive inflammation and/or fibrosis in IBD. Oncostatin M (OSM) which is an IL-6 family member and its receptor OSMR which is found mainly in stromal cells of the inflamed intestine have been associated with non-response to TNF [88]. When surgical resection samples from patients with IBD who either failed medical therapies or opted for elective surgery or required emergency surgery were analysed using bulk and single-cell RNA sequencing, histopathology and *in situ* localization different pathotypes were identified. One of the pathotypes was enriched with neutrophils and fibroblasts at sites of deep ulceration and was associated with non-response to anti-TNF, corticosteroids and anti-integrin therapy. IL-1 signalling via the IL-1 receptor on activated fibroblasts was found to be driving the influx of neutrophils at these sites [89].

A recent study which analysed biopsies taken from 38 patients with UC and CD and 3 healthy controls across five different gut regions before and after treatment with the TNF inhibitor adalimumab confirmed the heterogeneity in the stromal compartment and also identified inflammatory pathways within fibroblast populations that are associated with non-response to TNF [90]. The SOX6^+^ POSTN^+^ fibroblasts in the subepithelial region and ABCA8^+^ WNT2B^+^ FOS^hi^ and ABCA8^+^ WNT2B^+^ FOS^lo^ fibroblasts in the lamina propria showed increased expression of THY1, podoplanin (PDPN), OSMR, and of the neutrophil chemoattractants CXCL1 and CXCL6 after adalimumab treatment in patients with UC who did not achieve remission.

## Therapeutic strategies

It is now clear that fibroblasts change their phenotype in IMIDs which creates the formation of fertile soil for inflammation and/or fibrosis. Furthermore, the acquisition of epigenetic modifications and metabolic reprogramming seem to equip fibroblasts with ‘stromal memory’ which contributes to chronic and persistent inflammation. Fibroblasts have also been implicated in non-response to current therapies. It is therefore unsurprising that fibroblasts have become attractive therapeutic targets in IMIDs.

### Targeting fibroblast activation

There are different therapeutic strategies that could be adopted to target fibroblasts, some of which are already in clinical use. Blocking the activation of fibroblasts is one therapeutic approach. Fibroblasts are activated by many factors including TNF, IL-1 and TLR agonists [17]. TNF inhibitors block the activation of fibroblasts and of many other cell types including leukocytes and have revolutionized the treatment of RA and IBD. LIF, which drives the continued production of IL-6 in fibroblasts via an autocrine loop in RA [17], and OSM which is highly expressed in inflamed colonic tissues of patients with IBD and activates fibroblasts by binding to OSMR [88] are alternative therapeutic targets. CDH11, which is implicated in inflammation and cartilage degradation in RA, has also been examined as a therapeutic target. CDH11 is also upregulated in IBD compared with healthy colonic tissue and may have a role in intestinal fibrosis [91]. Blocking CDH11 via an mAb has been trialled in a phase II clinical trial, however there was no efficacy in patients with RA with an inadequate response to TNF inhibition [92].

### Targeting fibroblast derived molecules

Another therapeutic approach would be to target fibroblast derived molecules including cytokines, chemokines and MMPs. IL-6 inhibition is used in the treatment of many IMIDs including RA, GCA and Takayasu’s arteritis [93]. Blocking chemokines [94, 95] and MMPs [96, 97] has been trialled in clinical trials in patients with RA and has been less successful thus far.

### Targeting pathogenic fibroblast populations

**Figure 4:**
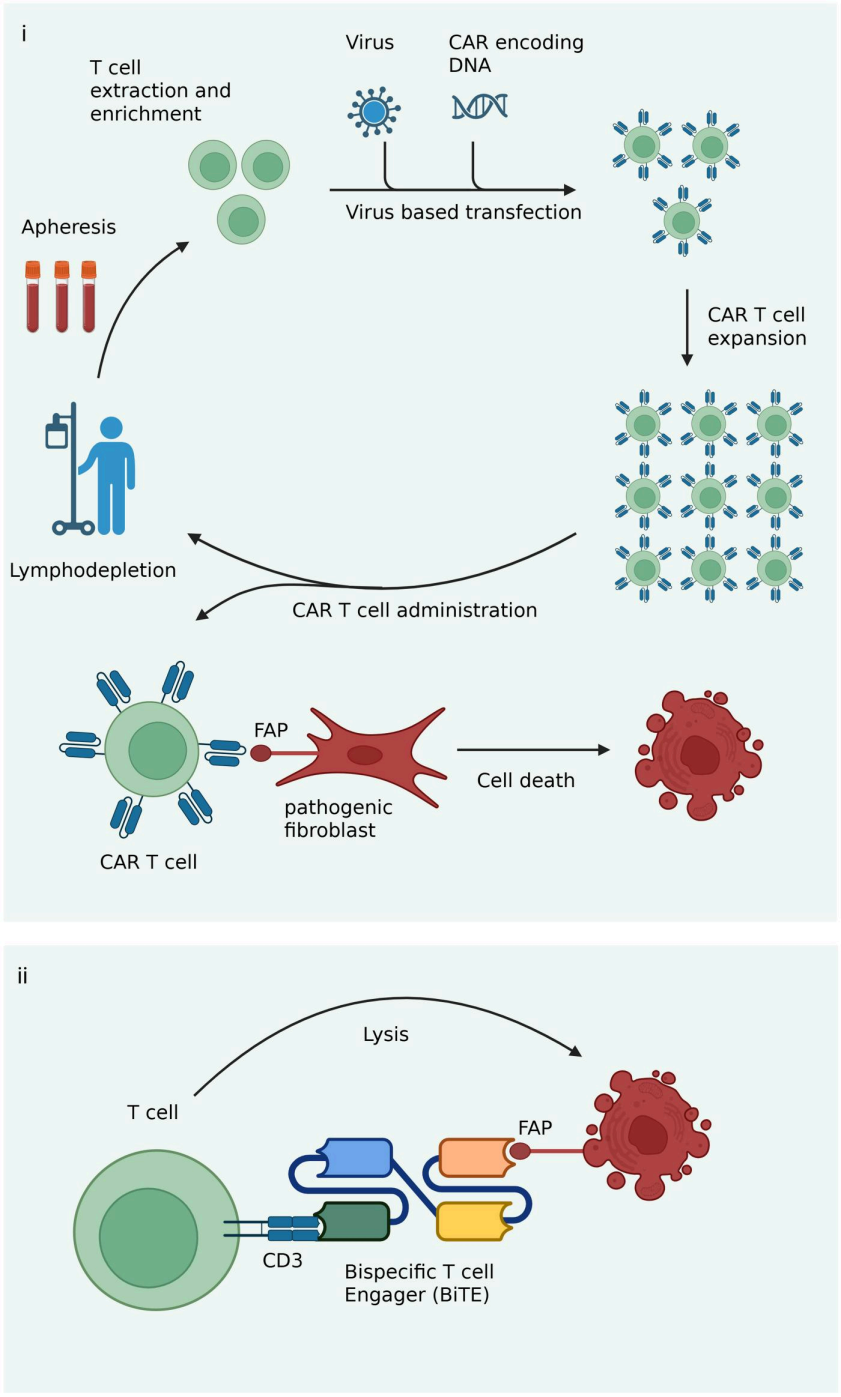
Targeting destruction of pathogenic fibroblast populations using Chimeric Antigen Receptor (CAR) T-cell therapy and T-cell engagers. (i) Lymphocytes extracted from patients’ peripheral blood are transfected with CAR-encoding DNA using a viral vector to generate CAR T cells. CAR T cells are expanded and administered to the patient. CAR T cells recognize the antigen, e.g. FAP on a pathogenic fibroblast upon infusion into the host, which leads to activation of the CAR T cell and targeted cell death. (ii) Bispecific T-cell engagers (BiTEs) consist of two immunoglobulin single-chain variable fragments connected with a flexible linker. They link T cells with a target cell, e.g. a pathogenic fibroblast, and mediate cell destruction via T-cell activation. Figure created using BioRender

Chimeric Antigen Receptor (CAR) T-cell therapies enable targeted destruction of pathogenic cell populations and are currently approved for the treatment of haematological malignancies (Fig. 4). They have also been trialled in SLE [98, 99] and UC [100]. CARs are composed of three domains; an extracellular domain which consists of a single chain variable-fragment (scFv) region, and a hinge, a transmembrane domain and an intracellular activation domain which contains the T cell receptor (TCR)-ζ and one or more co-stimulation domains. Lymphocytes extracted from patients’ peripheral blood are transfected with CAR-encoding DNA to generate CAR T cells. CAR T cells recognize the antigen upon infusion into the host which leads to activation of the CAR T cell and targeted cell death [101, 102]. The ability of CAR T cells to infiltrate deep into tissues and provide targeted cell-mediated toxicity gives hope that permanent resetting of the immune system in patients with autoimmune conditions is within sight. The use of CAR T cell therapy is, however, not without risks, including neurotoxicity and cytokine release syndrome (CRS) [103]. CD19-directed CAR T-cell therapy in a woman with severe treatment-refractory SLE led to complete clinical remission after 3 months and to the successful discontinuation of all immunosuppressive therapy without signs of disease relapse up to 18 months later [98]. *In vivo* CD19 CAR T-cell therapy which used CD8 T cell–targeted lipid nanoparticles encapsulating CD19 CAR mRNA have also been trialled in SLE [104]. The advantage of the *in vivo* approach is that it abrogates the need for lymphodepleting chemotherapy and is more cost and time efficient [104]. CAR T regulatory [105] and CAR NK [105, 106] cell therapies are also within sight.

Alternative approaches for targeted destruction of pathogenic fibroblast populations include T cell engagers (TCEs) (Fig. 4). TCEs are currently in use in oncology [99] and have been trialled in RA [107] and other autoimmune diseases [108, 109]. TCEs activate T cells to destroy target cells. Bispecific T-cell engagers (BiTEs) consist of two immunoglobulin scFv connected with a flexible linker. In blinatumomab, the first Food and Drug Administration–approved BiTE for acute lymphoblastic leukaemia [110], the N-terminal scFv recognizes CD19 on B cells and the C-terminal scFv binds the invariant CD3ε molecule on T cells [111]. Upon B-cell binding and T-cell engaging, the T cell induces apoptosis of the B cell via the release of perforin [110]. Blinatumomab was trialled in six patients with refractory RA and improved disease activity in all patients with no evidence of CRS. Importantly, there was depletion of memory B cells which were replaced by non-class-switched IgD+ naïve B cells, indicating an immune reset [107].

FAP is a promising target in CAR T-cell and TCE therapies in RA. FAP is a cell surface protein that is upregulated in lining and sublining fibroblasts in RA with minimal expression in healthy fibroblasts. CAR T-cell therapy against FAP has been trialled locally by intra-pleural injection in a phase I trial in malignant mesothelioma with no treatment toxicity [112]. CAR T-cell therapy against FAP has also been trialled in mice after cardiac injury and found to reduce cardiac fibrosis and improve cardiac function [113].

FAP is also expressed in CAFs and on some tumours themselves and has been targeted in oncology [114]. FAP-expressing cells have been targeted in oncology by delivering cytotoxic radiation to cells, using mAbs or small molecule inhibitors, or even with DNA vaccines. A notable example is ^177^Lu-dodecanetetraacetic acid tyrosine-3-octreotate (DOTATATE) which is approved for the treatment of neuroendocrine tumours [115]. ^177^Lu-FAPI04 was used in a study to assess its safety in patients with metastatic disease [116]. Sibrotuzumab, a human mAb to FAP was trialled in a phase II trial in patients with metastatic colorectal cancer; however, it did not have a therapeutic effect [117]. Talabostat, a small molecule inhibitor against FAP slowed tumour growth in mouse models of cancer and was subsequently trialled in a phase II clinical trial of metastatic colorectal cancer where there was no definite response to therapy, although 21% of patients had stable disease for 25 weeks [118]. DNA vaccines against FAP have also been trialled in murine models of metastatic colorectal cancer and been found to suppress tumour growth via CD8 T cell–mediated killing [119].

### Targeting the signalling pathway driving pathogenic fibroblast populations

Targeting the signalling pathway which leads to the formation of a pathogenic fibroblast population is also possible. This would have to be targeted early in disease prior to the expansion of the pathogenic population. One example is the NOTCH3 signalling pathway which drives the expansion of perivascular fibroblasts which drive inflammation in RA. Genetic deletion of NOTCH3 reduced inflammation and joint damage in a mouse model of arthritis [55]. Another example would be targeting the YAP/TAZ signalling pathway to prevent intestinal strictures in patients with CD [86].

### Targeting the epigenome and metabolome

Targeting the epigenome to reverse pathogenic fibroblast imprinting and the metabolic reprogramming of fibroblasts may disrupt ‘stromal memory’ and is another very appealing therapeutic approach. Restoring the stroma to a healthy state may allow the cessation of immunosuppressive treatment and gives hope for cure in IMIDs. C3 is an attractive therapeutic target which lies downstream of epigenetic modifications in fibroblasts and was shown to have an important role in tissue priming in RA [25]. Targeting the complement pathway has however been trialled in RA, and failed [120]. The inhibitory anti-C5 antibody eculizumab, a C5 antibody and C5aR inhibitor PMX-53 were unsuccessful in clinical trials of RA [120, 121]. Perhaps this may be explained by the fact that C5 lies further down the complement cascade. Targeting BET proteins is another potential therapeutic approach to targeting the epigenome in fibroblasts. BET protein inhibitors used *in vitro* suppressed the expression of many arthritogenic genes including MMPs and IL6 [73, 122].

Metabolic reprogramming and specifically a shift towards glycolysis secondary to C3a–C3aR activation in synovial fibroblasts was also found to be important in the tissue priming [25]. A shift towards glycolysis of synovial fibroblasts in RA has also been demonstrated in other studies [123]. Inhibition of glycolysis in a mouse model of arthritis was also found to reduce the severity of arthritis [123]. Inhibiting glycolysis is another therapeutic approach that can be adopted.

## Conclusion

Fibroblasts are heterogeneous cells that serve important functions in health and disease. Their heterogeneity is accompanied by a diverse functionality; with key roles in providing architectural support, defining tissue identity, and promoting inflammation, fibrosis and tissue destruction. Importantly, they display ‘stromal memory’ which may contribute to the persistence of inflammation. Despite their important role in IMIDs, there are no therapies that directly target fibroblasts. As the cellular basis of inflammation is uncovered and pathogenic fibroblast populations which contribute to inflammation and/or fibrosis are identified, targeted cell therapies give hope in ‘resetting’ the stroma back to health to treat, or even cure, these debilitating IMIDs.

## Data Availability

No new data were generated or analysed in this article.
